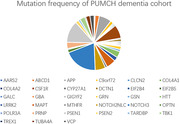# Genetic characteristics of PUMCH dementia cohort

**DOI:** 10.1002/alz.089001

**Published:** 2025-01-09

**Authors:** Liling Dong, Li Shang, Yuyue Qiu, Jialu Bao, Chenhui Mao, Shanshan Chu, Wei Jin, Bin Peng, Liying Cui, Jing Gao

**Affiliations:** ^1^ Peking Union Medical College Hospital, Beijing China

## Abstract

**Background:**

Dementia is prevalent over the world with unclear mechanism. Gene is supposed to play a vital role in the pathogenesis of dementia. However, the genetic basis of the majority of dementia patients is unknown.

**Method:**

All the participants were from the dementia cohort of Peking Union Medical College Hospital (PUMCH). They all received history inquiry, cognitive evaluation, biochemical test, brain imaging and gene testing. According to the standards of American College of Medical Genetics and Genomics (ACMG), the pathogenic/likely pathogenic variants (PLPV) related to dementia were screened.

**Result:**

2593 subjects were enrolled, including 1144 males and 1447 females. The average age was 66.8±12.1 years old. 56.3% (1461/2593) cases were early‐onset (age of onset < 65 years old), while 43.7% (1132/2593) cases were late onset. 66.4% (1722/2593) cases were APOE‐ε4 non‐carriers, and 33.6% (871/2593) cases were APOE‐ε4 carriers. 6.2% (162/2593) subjects carried the PLPV related to dementia. The NOTCH3 had the greatest mutation frequency (n=32, 1.2%), followed by the PSEN1 (n=21, 0.8%) and the CSF1R (n=13, 0.5%).

**Conclusion:**

There is genetic diversity among the PUMCH dementia cohort, with a high mutation frequency of the NOTCH3.